# Review of Mendelian randomization studies on common male-specific diseases

**DOI:** 10.3389/fendo.2025.1541744

**Published:** 2025-05-16

**Authors:** Qixin Pang, Zhe Chang, Hao Liu, Jianshe Chen, Sicheng Ma, Chenming Zhang, Zixue Sun

**Affiliations:** ^1^ Traditional Chinese Medicine (ZHONG JING) School, Henan University of Traditional Chinese Medicine, Zhengzhou, Henan, China; ^2^ School of Foreign Languages, Henan University of Traditional Chinese Medicine, Zhengzhou, Henan, China; ^3^ Queen Mary College, Nanchang University, Nanchang, Jiangxi, China; ^4^ Reproductive Medicine Department, Henan Province Hospital of Traditional Chinese Medicine, Zhengzhou, Henan, China; ^5^ The Second Clinical Medical School, Henan University of Traditional Chinese Medicine, Zhengzhou, Henan, China

**Keywords:** Mendelian randomization, male infertility, prostate cancer, erectile dysfunction, prostatitis

## Abstract

Although numerous Mendelian randomization studies on risk factors have been conducted in male medicine, a systematic synthesis of these findings is still lacking. This review searched relevant literature in PubMed and the Web of Science published before May 2024; systematically summarized the progress in the application of Mendelian randomization in male infertility, erectile dysfunction, prostate cancer, and prostatitis; summarized and classified the risk factors affecting men’s health, such as the gut microbiota, modifiable risk factors and related diseases; and presented some problems and solutions that were presented in these studies. This information offers valuable insights into the etiology and pathogenesis of male-specific diseases.

## 1 Introduction

Numerous medical statistics show that the incidence of male-specific diseases is increasing, and men’s health problems need urgent attention (1). Currently, the knowledge of risk factors associated with male-specific diseases needs to be further deepened. Mendelian randomization (MR) employs genetic variants highly correlated with exposure factors as instrumental variables (IVs) to ascertain the causal link between exposure and study outcomes. MR effectively reduces the impact of reverse causality and confounding factors. It also addresses the limitations of traditional medical statistics and epidemiological studies, offering a stronger foundation for identifying causal links between risk factors and disease risk (2). In recent years, many risk factor MR studies have been conducted in the field of male medicine, but there is a lack of systematic collation and summarization. In addition, a summary of the problems in the published literature is lacking. This article provides a systematic review of previous MR studies on risk factors for male-specific diseases, with the aim of providing ideas for the etiologic study and scientific prevention of male-specific diseases.

## 2 Methods

### 2.1 Fundamentals

**Figure 1:**
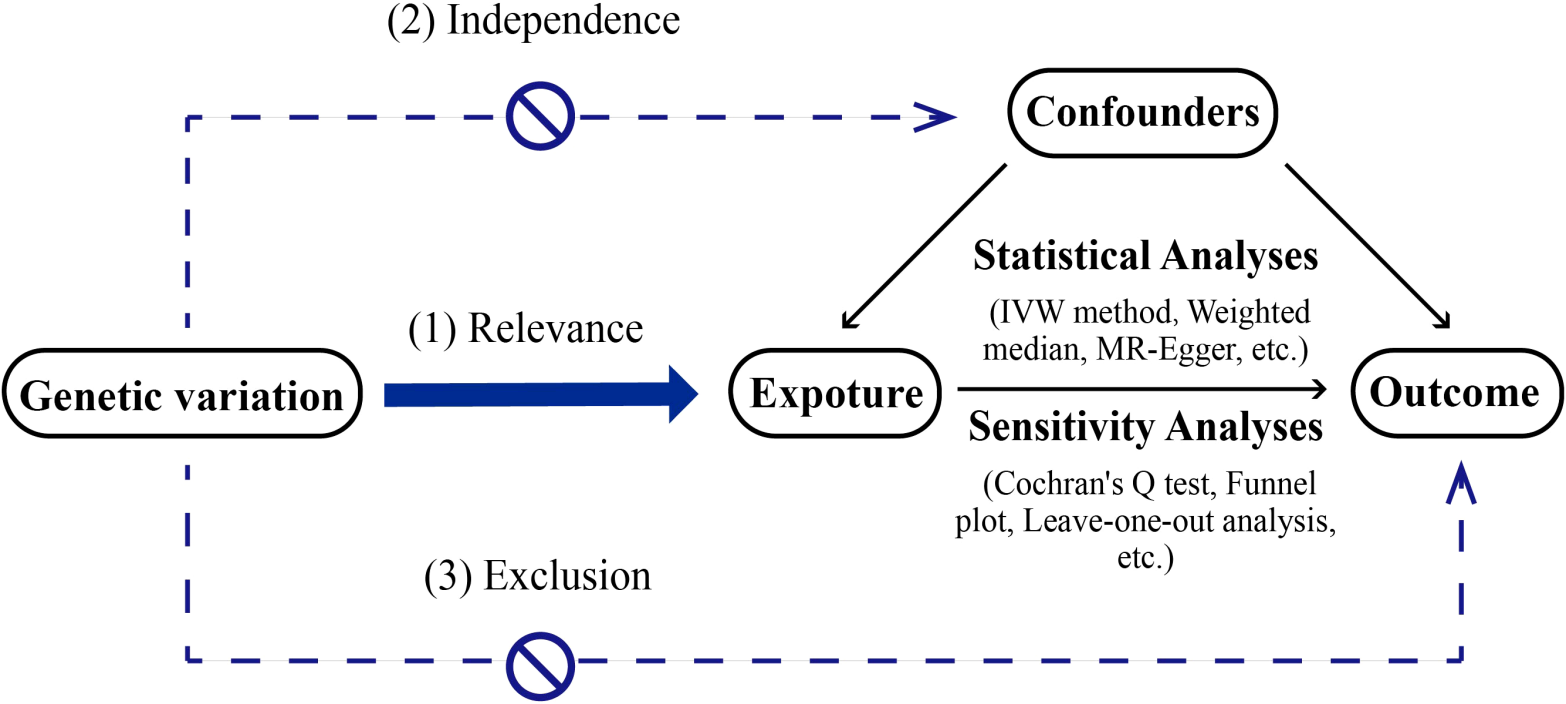
Diagram of mendelian randomization method.

MR serves as a methodological tool in scientific inquiry aimed at elucidating causal connections between exposure factors and outcomes. It operates by leveraging genetic variants that are strongly associated with exposure factors as IVs. Unlike conventional observational epidemiological studies, MR draws on the principles of Mendelian inheritance. This approach can be likened to a naturally occurring randomized controlled trial (RCT), albeit conducted within the framework of genetic inheritance. This method reduces the impact of confounding factors found in observational studies and offers strong evidence. MR studies need to follow 3 core assumptions (2, 3): (1) the assumption of association, meaning the instrumental variable is strongly linked to the exposure factor; (2) the assumption of independence, meaning the instrumental variable is not related to confounding factors; (3) the assumption of exclusivity, meaning the instrumental variable affects the outcome only through the exposure factor The description of MR method is shown in **Figure 1**.

### 2.2 Common types of MR methods

The study of MR can be divided into single-sample Mendelian randomization, two-sample Mendelian randomization (TSMR), multivariate Mendelian randomization (MVMR), two-step Mendelian randomization, bidirectional Mendelian randomization, etc.

Single-sample Mendelian randomization means that the association between genetic variations and exposure, as well as the correlation between genetic variations and outcomes, is obtained in the same sample. In this research method, a correlation exists between the regression coefficients of the numerator and denominator due to confounders between exposure and outcome, and weak instrumental bias can lead to overestimation of the exposure–outcome association (4).

Two-sample Mendelian randomization (TSMR) refers to obtaining two types of data, namely, relationships between genetic variations and exposure as well as relationships between genetic variations and outcomes, from two nonoverlapping datasets. This method mitigates the effects of weak instrumental bias and has greatly expanded the application scope of MR studies (5).

Multivariate Mendelian randomization (MVMR) considers the causal effects of multiple exposures on one or more outcome variables. It enables the simultaneous evaluation of various causal pathways and helps to resolve confounding among these factors (6).

Two-step Mendelian Randomization investigates potential mediating mechanisms linking risk factors to outcomes (7).

Bidirectional Mendelian Randomization is employed to validate causal directionality when the direction of causal association between a risk factor and outcome remains ambiguous (8).

### 2.3 Common statistical methods

Commonly used MR statistical analyses include inverse variance weighting (IVW), Weighted median, MR-Egger regression, MR-PRESSO, etc (9).

Inverse variance weighting (IVW) is the standard method used to aggregate MR data, which integrates summary data from multiple genetic variants, weighting individual causal effect estimates by inverse variances to provide consistent and efficient causal inference under valid IVs without linkage disequilibrium (10). The weighted median approach calculates a weighted median estimate of causal effects derived from multiple IVs, with weights assigned based on the inverse of each estimate’s sampling variance to prioritize precision. This method operates under the assumption that valid instruments collectively contribute over 50% of the total weighting scheme, ensuring robustness even in the presence of invalid IVs (11).

The MR-Egger method employs weighted regression to analyze the influence of associations between IVs and exposures on the associations between IVs and outcomes. This method incorporates an intercept term to quantify the average direct effect of IVs on the outcome, with its core assumption being the absence of correlated horizontal pleiotropy. To enhance the model’s adaptability, the method can be extended by including a random effects term, which is used to analyze the over-dispersion of causal effects across different IVs, thereby modeling pleiotropic variation (12).MR-PRESSO conducts a global assessment to detect potential outliers within an IVW framework, followed by a localized analysis to pinpoint specific outliers. The method further quantifies their impact through a distortion test evaluating systematic bias in causal effect estimates (13).

### 2.4 Disease selection strategy

Reference to the International Classification of Diseases (ICD-11) published by the World Health Organization (WHO) and authoritative urological literature (Campbell-Walsh-Wein Urology, volume 6) (14, 15), male diseases can be classified into the following categories: 1. Sexual health-related disorders: Including male infertility (MI), erectile dysfunction (ED), ejaculatory dysfunction (e.g., premature ejaculation [PE]), and sexually transmitted infections, etc. 2. Neoplasms of male genital organs: Such as prostate cancer (PCa), testicular tumors, and penile tumors. 3. Prostate diseases: Encompassing prostatitis, other prostatic disorders, and benign prostatic hyperplasia. 4. Structural abnormalities and congenital disorders of genitalia: Including hydrocele, testicular torsion, phimosis, and cryptorchidism, etc. 5. Inflammatory and infectious diseases: Such as orchitis and genital herpes. The selected 4 diseases in this article hold priority within the aforementioned disease categories:

1. For sexual health-related disorders, MI exhibits a high incidence rate. Globally, approximately 8-12% of couples suffer from infertility (16), with 50% of fertility issues attributable to male factors (17). Additionally, ED and PE are prevalent male sexual disorders in the general population. Population-based research indicates that 5% to 20% of males experience clinically significant ED (18), while PE occurs in about 30% of men aged 40–80 years (19). Despite PE being more prevalent than ED under the category of ejaculatory dysfunction, significant disparities in global incidence statistics and low healthcare-seeking rates among patients have resulted in far fewer research resources on PE compared to ED. Moreover, MR methodology-related studies on PE remain nearly absent. Therefore, this article selects ED as a research focus.
2. Among male genital organ tumors, PCa accounts for 29% of male cancers (20), ranking as the most common cancer among males in developed countries today (21). Given its representativeness in male genital tumors and abundant research data, PCa is emphasized in this article.
3. In prostate diseases, prostatitis is the most common urinary system disorder in males under 50 years old (15), while benign prostatic hyperplasia (BPH) represents the most prevalent benign tumor in elderly males (22). Comparatively, against the backdrop of population aging, research on BPH primarily stems from public health urgency, whereas prostatitis requires intensified mechanistic exploration due to its younger onset trends and chronic disease management challenges. MR methodology is particularly suitable for investigating the pathogenesis of prostatitis. Between these two conditions, this article prioritizes prostatitis as the study subject.
4. For structural/genital abnormalities and congenital diseases (e.g., hydrocele, testicular torsion, phimosis, and cryptorchidism), their relatively low incidence precludes their selection in this study. Among inflammatory and infectious diseases, prostatitis is chosen as a representative condition.

In summary, considering the article’s scope limitations, this review focuses on 4 diseases characterized by high incidence rates, broad societal impact, significant impairment of male patients’ quality of life, complex etiological mechanisms, and substantial existing literature.

### 2.5 Risk factor selection and classification strategy

This study employed a three-tiered criteria for risk factor selection: First, integration of authoritative guidelines and consensus statements, including the World Health Organization (WHO) framework for noncommunicable disease risks, the European Association of Urology (EAU) Guidelines on Sexual and Reproductive Health, and etiological evidence from classical urological literature (15). Second, prioritization of factors with high evidence strength validated by large-scale cohort studies (e.g., smoking, sedentary behavior) to ensure conclusion reliability. Third, focus on clinically actionable risk indicators, particularly lifestyle-related factors (e.g., dietary patterns, exercise habits), as these can be modulated through public health policies or individual behavioral adjustments. This strategy balances scientific rigor with practical translational value, providing multidimensional evidence for male reproductive health management.

Based on these criteria, the risk factors included in this study are categorized into 6 types: Gut microbiota, circulatory substance (cytokines), related diseases, modifiable risk factors, drug targets, other risk factors (limited studies or irrelevant factors).

### 2.6 Search strategy and selection criteria of references

Original studies were identified by searching relevant articles in the PubMed and Web of Science databases through May 2024.The following terms were used to search: “mendelian randomization” or “genetic instrumental variable” or “genetic instrument”, “male infertility” or “male sterility”, “prostate cancer” or “prostatic carcinoma” or “prostatic cancer”, “erectile dysfunction” or “impotence”, “prostatitis” or “prostate inflammation”, etc. Inclusion criteria: (1) Mendelian Randomization (MR) study design; (2) Genetic variants or Genetic Risk Scores (GRS) were used as Instrumental Variables (IVs) to analyze the relationship between exposure and outcome. Articles were excluded for the following reasons: reviews, non-original articles, non-human studies, study protocols, letters, conference abstracts, and articles for which the full text is not available. We finally included 122 articles and categorized them according to disease type, as shown in **Tables 1**–**4**.

**Table 1:** Application of Mendelian randomization in male infertility.

Disease	Reference no.	Exposure	SNPs, n	OR (95%CI)	P-value	Sample size		Correlation	Population	MR method
						Cases	Control			
**Male infertility**	PMID: 36235694	BMI	35	1.24 (1.09-1.40)	0.001	680	72,799	Risk factor	European	IVW
		Body fat percentage	499	1.73 (1.13-2.64)	0.011			Risk factor		
		Alcohol consumption	50	6.58 (1.20-36.14)	0.03			Risk factor		
	PMID: 34668019	BMI	896	1.26 (1.08-1.48)	0.003	26,252		Causal role		IVW
	PMID: 35562204	Smoking	378	1.10 (0.78-1.56)	0.179	3,275	23,821	No robust evidence	European	IVW
	PMID: 38715795	Coffe intake	40	3.70 (1.03-13.21)	0.044	680	72,799	Positive correlation	European	
		Cooked vegetable intakes	17	54.79 (2.90-1030.55)	0.008			Positive correlation		
	PMID: 38711980	Anaerotruncus	13	1.81 (1.18-2.77)	0.006	1,271	119,297	Increased the risk	European	IVW
		Bacteroides	8	0.57 (0.33-0.96)	0.036			Decreased the risk		
		HGF	7	0.50 (0.35-0.71)	<0.001			Reduced the risk		
		MCP-3	5	1.29 (1.09-1.53)	0.004			Increased the risk		
	PMID: 38489097	Bacteroidaceae	8	0.54 (0.31-0.96)	0.035	1,271	119,297	Decreased the risk	European	IVW
		Bacteroides	8	0.54 (0.31-0.96)	0.035			Decreased the risk		
		RuminococcaceaeNK4A214group	13	0.56(0.36-0.89)	0.014			Decreased the risk		
		RuminococcaceaeUCG011	8	0.76 (0.59-0.99)	0.042			Decreased the risk		
		Anaerotruncus	13	1.96 (1.13-3.40)	0.016			Increased the risk		
	PMID: 37822739	Anaerotruncus	13	1.96 (1.13-3.40)	0.016	1,128	110,070	Positively associated	European	IVW
		Bacteroides	9	0.58 (0.34-0.99)	0.048			Negatively associated		
		Ruminococcaceae (NK4A214group)	13	0.57 (0.36-0.89)	0.014			Negatively associated		
		Ruminococcaceae (UCG011)	8	0.76 (0.59-0.99)	0.042			Negatively associated		
		Romboutsia	13	0.67 (0.43-1.03)	0.067			Negatively correlated		
		Lactococcus	9	1.29 (0.97-1.72)	0.085			Positively associated		
		Allisonella	8	1.28 (0.96-1.71)	0.091			Positive		
		Anaerotruncus	13	1.96 (1.13-3.40)	0.016			Positive		
		Intestinibacter	15	1.51 (0.96-2.39)	0.074			Positive		
		Anaerofilum	10	0.70 (0.46-1.07)	0.097			Negative		
		Barnesiella	14	1.41 (0.86-2.31)	0.175			Positive		
	PMID: 37764164	Bacteroidaceae	8	0.57 (0.33-0.96)	0.036	1,271	119,297	Positive	European	IVW
		Bacteroides	8	0.57 (0.33-0.96)	0.036			Positive		
		Ruminococcaceae NK4A214 group	13	0.61 (0.39-0.97)	0.037			Positive		
		Anaerotruncus	13	1.81 (1.14-2.87)	0.011			Causal association		
	PMID: 37454180	Allisonella	8	1.32 (1.02-1.72)	0.03	994	100,050	Positive	European	IVW
		Bacteroidaceae	7	0.44 (0.23-0.83)	0.01			Negative		
		Bacteroides	7	0.44 (0.23-0.83)	0.01			Negative		
		Enterobacteriales	7	0.47 (0.23-0.95)	0.03			Negative		
		Romboutsia	14	0.64 (0.42-0.96)	0.03			Negative		
		Enterobacteriaceae	7	0.47 (0.23-0.95)	0.03			Negative		
	PMID: 38619404	Pasteurellales	17	0.67 (0.47-0.94)	0.022	680	72,799	Decreased the risk	European	IVW
		Bacteroidaceae	12	0.49 (0.27-0.90)	0.022			Decreased the risk		
		Pasteurellale	17	0.67 (0.47-0.94)	0.022			Decreased the risk		
		Bacteroide	12	0.49 (0.27-0.91)	0.022			Decreased the risk		
		Eubacterium rectale group	12	0.45 (0.26-0.78)	0.004			Decreased the risk		
		RuminococcaceaeNK4A214group	16	0.55 (0.32-0.95)	0.033			Decreased the risk		
	PMID: 37605651	Eubacterium oxidoreducens	5	2.05 (1.20-3.49)	0.008	680	72,799	Risk factor	European	IVW
		Lactococcus	9	1.45 (1.01-2.06)	0.042			Risk factor		
		Eubacterium ventriosum	9	0.44 (0.22-0.87)	0.018			Protective factor		
		Eubacterium rectale	8	0.31 (0.15-0.64)	0.002			Protective factor		
		Ruminococcaceae NK4A214	13	0.54 (0.29-0.99)	0.045			Protective factor		
	PMID: 36593707	25OHD	99	0.62 (0.44-0.89)	0.01	825	85,722	Protective factor	European	IVW
	PMID: 38479056	HGF	6	3.77 (1.80-7.91)	0.0004	1,271	11,9297	Positively associated	European	IVW
		IL-2ra	17	1.29 (1.11-1.49)	0.001			Positively associated		
		RANTES	1	2.59 (1.37-4.91)	0.003			Positively associated		Wald ratio
		SCF	5	0.40 (0.18-0.88)	0.023			Positively associated		
	PMID: 38152129	T2DM	58	0.77 (0.60-0.98)	0.034	680	72,799	Significant causal relationship	European	IVW
	PMID: 38529400	T2DM	62	0.82 (0.70-0.97)	0.017	680	72,799	Substantial causal relationship	European	IVW
	DOI: 10.22514/jomh.2024.009	COVID-19	12	0.86 (0.65-1.15)	0.308	680	72,799	No causal effect	European	IVW
	PMID: 38457599	COVID-19	5	0.47 (0.16-1.41)	0.178	680	72,799	No clear causal relationship	European	IVW
	PMID: 38814907	Ulcerative colitis	86	1.13 (1.00-1.26)	0.046	680	72,799	Positive	European	IVW
	PMID: 38699446	Mood disorders	35	1.45 (1.01-2.08)	0.044	680	72,799	Positive	European	IVW
		Attention deficit hyperactivity disorder	30	0.83 (0.34-2.02)	0.686			Positive		
		Obsessive-compulsive disorder	13	0.93 (0.68-1.26)	0.637			Negative		
	PMID: 38456015	Chronotype	147	0.88 (0.42-1.83)	0.725	680	72,799	No evidence	European	IVW
		Sleep duration	64	0.99 (0.26-3.77)	0.994			No evidence		
		Insomnia	38	0.34 (0.05-2.49)	0.29			No evidence		
		Snoring	39	0.53 (0.03-9.40)	0.667			No evidence		
		Dozing	30	3.62 (0.19-70.21)	0.395			No evidence		
		Daytime nap	93	2.64 (0.67-10.40)	0.164			No evidence		
		Oversleeping	31	1.55 (0.25-9.58)	0.635			No evidence		
		Undersleeping	25	4.28 (0.44-43.22)	0.206			No evidence		
	PMID: 38512957	LTL	135	1.27 (0.84-1.92)	0.261	680	72,799	No causal associations	European	IVW
	PMID: 34778177	Educational Attainment	1,271	0.79 (0.52-1.20)	0.269	680	72,799	Not related	European	IVW

**Table 2:** Application of Mendelian randomization in erectile dysfunction.

Disease	Reference no.	Exposure	SNPs, n	OR	P-value	Sample size		Correlation	Population	MR method
						Cases	Control			
**Erectile dysfunction**	PMID: 36844727	Coronary heart disease	43	1.09 (1.01-1.18)	0.022	6,175	217,630	Increase the risks	European	IVW
		Heart failure	9	1.36 (1.07-1.74)	0.013			Increase the risks		
		Ischemic heart disease	31	3.22 (0.64-16.22)	0.156			No causal association		
		Atrial fibrillation	139	1.03 (0.97-1.08)	0.312			No causal association		
	PMID: 36891666	IS	9	1.34 (1.08-1.21)	0.007	6,175	223,805	Causally associated	European	IVW
		HF	9	1.36 (1.07-1.74)	0.013			Causally associated		
		Coronary heart disease	43	1.15 (1.09-1.18)	0.022			Causally associated		
	PMID: 37363097	Hypertension	154	1.10 (1.02-1.20)	0.017	6,175	217,630	Increases the risk	European	IVW
	PMID: 37025676	Hypertension	67	3.83 (2.30-6.38)	0.0085	6,175	223,805	Positive causal link	European	IVW
	DOI: 10.22514/jomh.2023.084	High blood pressure	149	1.66 (1.13-2.45)	0.001	6,175	223,805	Increased odds	European	IVW
	PMID: 37782322	Coronary artery disease	88	1.09 (1.02-1.16)	0.013	6,175	217,630	Causally associated	European	IVW
		Coronary heart disease	42	1.07 (1.01-1.13)	0.017			Causally associated		
		Myocardial infection	87	1.09 (1.02-1.17)	0.011			Causally associated		
		Atrial fibrillation	216	1.06 (1.00-1.12)	0.04			Causally associated		
	PMID: 37833702	HF	30	1.17 (0.99-1.39)	0.074	6,175	217,630	No significant causal relationship	European	IVW
		Coronary heart disease	61	1.08 (0.99-1.17)	0.068			No significant causal relationship		
	PMID: 36891666	IS	9	1.34 (1.08-1.66)	0.007	6,175	223,805	Causally associated	European	IVW
		HF	9	1.36 (1.07-1.74)	0.013			Causally associated		
		Coronary heart disease	43	1.09 (1.01-1.18)	0.022			Causally associated		
	PMID: 34842357	T2DM	137	1.15 (1.05-1.25)	0.001	6,175	223,805	Direct causal effect	European	IVW
	PMID: 30583798	T2DM	103	1.11 (1.05-1.17)	3.50E-04	6,175	223,805	Causally implicated	European	IVW
	PMID: 36313469	Depression	73	1.68 (1.38-2.05)	< 0.001	6,175	223,805	Increases the incidence	European	IVW
	PMID: 36997981	Major depression	37	1.53 (1.19-1.96)	0.001	6,175	223,805	Causally related	European	IVW
		Bipolar disorder	34	0.95 (0.87-1.04)	0.36			No causal impact		
	PMID: 37541893	Major depressive disorder	44	1.32 (1.08-1.62)	< 0.001	6,175	217,630	Higher risk	European	IVW
	PMID: 35819009	COVID-19	6	1.24 (1.04-1.46)	< 0.05	6,175	217,630	Elevated risk	European	IVW
	PMID: 38085233	COVID-19	91	1.07 (1.01-1.13)	< 0.06	6,175	217,630	Correlated	European	IVW
	PMID: 37415973	COVID-19	7	1.09 (1.03-1.16)	0.004	6,175	217,630	Increased risk	European	IVW
	PMID: 38264202	Ulcerative colitis	37	0.96 (0.91-1.01)	0.08	6,175	223,805	No evidence	European	IVW
		Crohn's disease	54	1.04 (0.99-1.08)	0.091			No evidence		
	PMID: 38784037	IBD	62	1.11 (1.02-1.21)	0.019	1,154	94,024	Increased risk	European	IVW
		Ulcerative colitis	35	1.02 (0.92-1.14)	0.679			No significant evidence		
		Crohn's disease	51	1.09 (1.02-1.17)	0.014			Increased risk		
	PMID: 38272986	IBD	62	1.11 (1.02-1.21)	0.019	1,154	94,024	Increased the incidence	European	IVW
		Crohn's disease	52	1.09 (1.02-1.16)	0.016			Increased the incidence		
		Ulcerative colitis	36	1.02 (0.92-1.13)	0.743			No causal effect		
	PMID: 37928685	Lachnospiraceae	17	1.27 (1.05-1.52)	0.012	6,175	217,630	Risk factor	European	IVW
		Senegalimassilia	5	1.32 (1.06-1.64)	0.012			Risk factor		
		Lachnospiraceae NC2004 group	8	1.20 (1.01-1.41)	0.03			Risk factor		
		Tyzzerella3	13	1.14 (1.02-1.27)	0.024			Risk factor		
		Oscillibacter	13	1.20 (1.04-1.39)	0.016			Risk factor		
		Ruminococcaceae UCG013	12	0.77 (0.62-0.97)	0.023			Protective effect		
	PMID: 38311371	Lachnospiraceae	27	1.27 (1.05-1.52)	0.01	6,175	223,805	Higher risk	European	IVW
		LachnospiraceaeNC2004 group	10	1.17 (1.01-1.37)	0.04			Higher risk		
		Oscillibacter	17	1.17 (1.02-1.35)	0.03			Increase the risk		
		Senegalimassilia	8	1.32 (1.06-1.64)	0.01			Increase the risk		
		Tyzzerella3	14	1.14 (1.02-1.27)	0.02			Increase the risk		
		RuminococcaceaeUCG013	14	0.77 (0.61-0.96)	0.02			Protective effect		
	PMID: 38390206	LDL Receptor agonists	42	0.76 (0.56-0.95)	0.005	6,175	223,805	Reduced risk	European	IVW
		Lipoprotein Lipase agonists	56	0.91 (0.78-1.04)	0.138			Reduced risk		
		Apolipoprotein C-III inhibitors	37	0.90 (0.77-1.02)	0.087			Reduced risk		
		Apolipoprotein B-100 inhibitors	29	1.03 (0.75-1.32)	0.816			Elevated risk		
	PMID: 38741592	Atorvastatin use		23.91	0.02	6,175	217,630	Increased risk	European	IVW
	PMID: 38260164	Aspirin use	9	20.896 (2.077-210.2)	0.01	6,175	217,630	Predisposing factor	European	IVW
	PMID: 38827362	Fibroblast growth factor 5	503	1.05 (1.01-1.11)	0.0307	2,205	164,104	Increased risk	European	IVW
		IL-22 receptor subunit alpha-1	21	1.28 (1.01-1.62)	0.0406			Increased risk		
		Protein S100-A12	23	1.22 (1.02-1.47)	0.0314			Increased risk		
		TNF-related activation-induced cytokine	43	0.88 (0.78-0.99)	0.048			Decreased risk		
	PMID: 38680495	Interferon-inducible protein-10	9	1.27 (1.01-1.60)	0.043	2,205	164,104	Elevate the risk	European	IVW
		Interleukin-1 receptor antagonist	8	0.77 (0.60-0.98)	0.037			Reduce the risk		
	PMID: 37236543	BMI	834	1.23 (1.11-1.37)	<0.001	6,175	217,630	Increased risk	European	IVW
		Waist circumference	278	1.30 (1.13-1.49)	<0.002			Increased risk		
		Trunk fat mass	632	1.13 (1.01-1.36)	0.035			Increased risk		
		Whole body fat mass	630	1.18 (1.06-1.37)	0.003			Increased risk		
	PMID: 37082877	BMI		1.84 (1.05-1.36)	0.006	6,175	223,805	Increased risk	European	IVW
	PMID: 35692403	Snoring	19	3.45 (1.68-7.09)	<0.001	6,175	217,630	Increased risk	European	IVW
	PMID: 38505341	Ever smoked	16	5.89 (1.60-21.94)	0.01	6,175	217,630	Increased risk	European	IVW
		Alcohol consumption	38	1.50 (1.05-2.14)	0.03			Increased risk		
		BMI	444	1.18 (1.06-1.31)	0.003			Increased risk		
		Earlier age at first intercours	260	0.66 (0.55-0.78)	2.50E-06			Reduced risk		
	PMID: 35946227	Insomnia	196	1.15 (1.07-1.23)	<0.001	6,175	217,630	Increased the risk	European	IVW
	PMID: 38131625	Insomnia	33	3.44 (1.59-7.44)	0.001	6,175	217,630	Higher risk	European	IVW
	PMID: 33548002	TSH	60	-0.00 (-0.001, 0.001)	0.914	166,988		No evidence	European	IVW
	PMID: 37581767	Periodontal disease	6	1.07 (0.96-1.20)	0.22	6,175	217,630	No evidence	European	IVW

**Table 3:** Application of Mendelian randomization in prostate cancer.

Disease	Reference no.	Exposure	SNPs, n	OR (95%CI)	P-value	Sample Size		Correlation	Population	MR method
						Cases	Control			
**Prostate cancer**	PMID: 31802111	Physical activity	2	0.49 (0.33-0.72)	3.00E-04	79,148	61,106	Inversely associated	European	IVW
		Serum iron levels	5	0.92 (0.86-0.98)	0.007			Inversely associated		
		BMI	535	0.90 (0.84-0.97)	0.003			Inversely associated		
		Circulating monounsaturated fat	5	1.11 (1.02-1.20)	0.02			Circulating monounsaturated fat		
	PMID: 26387087	Increased height	179	0.99 (0.96-1.00)	0.23	20,848	20,214	Not associated	European	GRS+ Logistic regression
		BMI	32	0.98 (0.97-1.01)	0.07			Decreased risk		
	PMID: 37305903	Unfavourable adiposity	27	0.85 (0.61-1.19)	0.35	85,554	91,972	No strong evidence	European	IVW
		Favourable adiposity	34	0.80 (0.53-1.23)	0.32			No strong evidence		
		BMI	506	0.97 (0.88-1.08)	0.59			No strong evidence		
	PMID: 32701947	Smoking	361	0.90 (0.80-1.02)	0.104	79,148	61,106	Nonsignificant inverse association	European	IVW
	PMID: 37237487	Smoking	108	1.95 (1.09-3.50)	0.027	79,148	61,106	Risk factor	European	IVW
	PMID: 33193711	Shorter LTL	17	0.94 (0.91-0.98)	0.005	27,641	307,395	Decreased risk	European	IVW
	PMID: 31981976	Shorter LTL	10	HR:1.73 (1.08-2.78)	0.021	1,889		High Gleason scores, worse prognosis	European	IVW
	PMID: 37352282	Longer LTL	134	1.37 (1.25-1.50)	2.84E-11	79,148	61,106	Increased risk	European	IVW
	PMID: 31089709	MSP	1	0.65 (0.51-0.84)	0.001	1,871	1,871	Inversely associated	European	IVW
	PMID: 38594418	Zinc	3	1.06 (1.01-1.12)	0.026	79,194	61,112	Weak causal effect	European	IVW
	PMID: 36923697	Zinc	2	1.06 (1.00-1.12)	0.04	79,148	61,106	Increased risk	European	IVW
	PMID: 34617559	Phosphorus	125	1.19 (1.09-1.31)	1.82E-04	79,148	61,106	Increased risk	European	IVW
	PMID: 36561528	Iron	3	0.91 (0.84-0.99)	0.035	79,148	61,106	Decreased risk		IVW
	PMID: 35085228	lipoprotein A	10	1.07 (0.91-1.25)	0.431	79,166	61,106	Increased risk	European	IVW
	PMID: 26992435	LDL	11	1.50 (0.92-2.46)	0.11	22,249	22,133	Weak evidence	European	GRS+ Logistic regression
	PMID: 36595504	PCSK9	28	0.85 (0.76-0.96)	0.009	79,194	61,112	Lower risk	European	IVW
	PMID: 35151363	PCSK9	8	0.81 (0.73-0.90)	4.52E-05	79,148	61,106	Reduced risk	European	IVW
	PMID: 36316671	Triglyceride	48	1.002 (1.000-1.004)	0.016	3436	459,574	Increased risk	European	IVW
	PMID: 35296245	Aspartate	4	1.04 (1.00-1.08)	0.034	79,148	61,106	Positively associated	European	IVW
	PMID: 36330075	Alanine	16	1.16 (1.01-1.33)	0.037	79,148	61,106	Increased risk	European	IVW
		Aminotransferase	237	0.43 (0.27-0.68)	3.28E-04			Inversely associated		
	DOI: 10.21203/rs.3.rs-2815251/v1	Mean corpuscular volume	378	0.95 (0.90-0.98)	0.004	79,148	61,106	Decreased risk	European	IVW
		Mean corpuscular hemoglobin	366	0.94 (0.91-0.99)	0.019			Decreased risk		
		Mean corpuscular hemoglobin concentration	102	0.89 (0.81-0.98)	0.023			Decreased risk		
	PMID: 35012533	Macrophage inflammatory protein 1a	35	1.06 (1.03-1.10)	5.62E-04	79,148	61,106	Positive association	European	IVW
		Vascular endothelial growth factor	21	0.86 (0.79-0.93)	2.28E-04			Inverse association		
	PMID: 36733309	IL-6	2	1.12 (1.07-1.17)	6.61E-07	79,148	61,106	Increased risk	European	IVW
		IL-1ra	4	0.92 (0.89-0.96)	1.58E-05			Reduced risk		
	PMID: 36482455	Bioavailable testosterone	52	1.17 (1.09-1.26)	2.51E-05	79,148	61,106	Increased risk	European	IVW
	PMID: 35579976	Testosterone	67	1.23 (1.08-1.40)	0.002	79,148	61,106	Positive	European	IVW
	PMID: 38867724	Proinsulin	48	0.94 (0.89-0.999)	0.048	79,148	61,106	Negative factor	European	IVW
	PMID: 38911377	Sodium-glucose Cotransporter 2 Inhibition	6	1.17 (0.59-1.74)	<0.001	79,194	61,112	Increased risk	European	IVW
	PMID: 38701318	HMGCR	12	1.62 (1.23-2.12)	0.0005	211,227		Elevated risk	European	IVW
	PMID: 38517045	Genetically proxied metformin effects	13	1.55 (1.23-1.96)	0.003	79,148	61,106	Increased risk	European	IVW
	PMID: 35151363	Genetically proxied inhibition of PCSK9	11	0.81 (0.73-0.90)	4.52E-05	79,148	61,106	Negatively associated	European	IVW
	PMID: 38487860	Drugs	105	0.94 (0.91-0.97)	7.00E-04	79,148	61,106	Reduced risk	European	IVW
	PMID: 37735436	KDELC2	1	0.89 (0.86-0.93)	1.89E-08	79,148	61,106	Negatively associated	European	IVW
	PMID: 33032658	Prevotella	1	-0.758 (-1.354, -0.162)	0.013	495	640	Decrease	East Asia	IVW
	PMID: 36880394	Class Alphaproteobacteria	7	0.84 (0.75-0.93)	0.001	79,148	61,106	Negatively associated	European	IVW
	PMID: 38369514	Odoribacter	7	1.17 (1.05-1.31)	0.005	79,148	61,106	Higher risk	European	IVW
		Dorea	9	1.13 (1.02-1.25)	0.025			Higher risk		
		Christensenellaceae R7	9	1.12 (1.01-1.25)	0.032			Higher risk		
		Eubacterium fissicatena	9	1.08 (1.02-1.13)	0.006			Higher risk		
		Ruminococcus gauvreaui	12	1.10 (1.01-1.19)	0.032			Higher risk		
		Eubacterium nodatum	11	1.06 (1.02-1.11)	0.007			Higher risk		
		Lachnospiraceae	17	1.08 (1.00-1.16)	0.046			Higher risk		
		Flavonifractor	5	0.84 (0.75-0.94)	0.003			Lower risk		
		Adlercreutzia	8	0.89 (0.82-0.97)	0.005			Lower risk		
		Roseburia	14	0.90 (0.83-0.98)	0.019			Lower risk		
		Ruminococcaceae UCG004	9	0.91 (0.84-0.99)	0.027			Lower risk		
		Coprobacter	11	0.92 (0.87-0.98)	0.008			Lower risk		
		Allisonella	6	0.93 (0.89-0.99)	0.014			Lower risk		
		Holdemania	15	0.93 (0.88-0.99)	0.014			Lower risk		
		Rhodospirillaceae	15	0.94 (0.89-1.00)	0.037			Lower risk		
		Rhodospirillales	14	0.91 (0.86-0.97)	0.003			Lower risk		
		Alphaproteobacteria	7	0.84 (0.76-0.92)	<0.001			Lower risk		
	PMID: 37697271	Allisonella	1	0.89 (0.81-0.99)	0.038	79,148	61,106	Decreased risk	European	IVW
	PMID: 38029073	Akkermansia muciniphila	5	0.79 (0.67-0.94)	0.009	6,311	88,902	Negatively associated	European	IVW
		Bacteroides salyersiae	6	0.90 (0.83-0.99)	0.022			Negatively associated		
		Eubacterium biforme	4	1.16 (1.01-1.34)	0.035			Positively associated		
	PMID: 37274339	Genetically predicted hyperthyroidism	13	0.86 (0.79-0.93)	4.00E-04	6,321	354,873	Declining risk	European	IVW
	PMID: 37213031	Systemic lupus erythematosus	48	0.98 (0.97-0.99)	0.003	79,148	61,106	Decreased risk	European	IVW
	PMID: 38783043	Systemic lupus erythematosus	4	0.94 (0.91-0.97)	2.14E-04	79148	61106	Lower risk	European	IVW
		Hyperthyroidism	25	0.02 (0.0016-0.2539)	0.003			Lower risk		
		Rheumatoid arthritis	129	1.03 (1.02-1.05)	2.13E-05			Developing		
	PMID: 38703296	Obstructive sleep apnea	5	0.87 (0.79-0.95)	0.002	79,148	61,106	Negatively associated	European	IVW
	PMID: 38741062	Pernicious anemia	17	-0.022 (-0.035 , -0.006)	0.007	6,311	74,685	Reverse causal relationship	European	IVW
	PMID: 38403547	Erysipelas	28	1.05 (1.01-1.08)	0.005	6,311	74,685	Significant association	European	IVW
	PMID: 35303584	Schizophrenia	75	1.033 (0.998-1.069)	0.065	79,148	61,106	Not support	European	IVW
	PMID: 33027558	Depression	44	0.72 (0.35-1.47)	0.364	79,148	61,106	No strong evidence	European	IVW
	PMID: 31908803	Fasting glucose	21	0.93 (0.73-1.17)	I²: 0.46	79,148	61,106	No association	European	IVW
		HbA1c	34	0.90 (0.58-1.40)	I²: 0.58			No association		
		Type 2 diabetes	159	1.02 (0.97-1.07)	I²: 0.69			No association		
	PMID: 32349989	Type 2 diabetes	399	0.97 (0.93-1.01)	0.108	7,872	359,711	No association	European	IVW
	PMID: 27598322	Adult height	168	1.03 (0.92-1.15)	0.642	14,160	12,724	No association	European	IVW
	PMID: 35906597	Circulating vitamin E	3	0.85 (0.59-1.23)	0.388	79,148	61,106	No association	European	IVW
	PMID: 34325683	Circulating vitamin C	11	0.90 (0.74-1.09)	0.29	79,148	61,106	No association	European	IVW
	PMID: 33420236	Circulating vitamin D	138	-0.02 (-0.09, -0.05)	0.57	79,148	61,106	No association	European	IVW
	PMID: 34504857	Homocysteine	15	1.01 (0.93-1.11)	0.774	79,148	61,106	No association	European	IVW
	PMID: 35494045	Tryptophan	18	-0.92 (-2.04, 0.20)	0.11	79,148	61,106	Not significantly associate	European	IVW
	PMID: 37178364	Systolic blood pressure	278	0.96 (0.92-1.01)	0.11	79,148	61,106	No strong evidence	European	IVW
		Blocking calcium channel receptors	16	1.22 (1.06-1.42)	0.01			Increased risk		
	PMID: 33805346	Serum urea	6	1.02 (0.94-1.11)	0.703	79,148	61,106	Null association	European	IVW
	PMID: 32006205	Allergic diseases	132	1.00 (0.94-1.05)	0.93	79,148	61,106	No evidence	European	IVW
	PMID: 33671849	Circulating Bilirubin Levels	115	1.00 (0.97-1.03)	1	79,194	61,112	No evidence	European	IVW
	PMID: 36204379	Processed meat	23	1.02 (0.69-1.49)	0.94	79,148	61,106	No evidence	European	IVW
	PMID: 33199044	Arachidonic acid	5	1.02 (1.00-1.04)	0.114	79,148	61,106	No evidence	European	IVW
	PMID: 33178578	C-reactive protein	58	1.06 (0.96-1.16)	0.24	79,148	61,106	No evidence	European	IVW

**Table 4:** Application of Mendelian randomization in prostatitis.

Disease	Reference no.	Exposure	SNPs, n	OR (95%CI)	P-value	Sample size		Correlation	Population	MR method
						Cases	Control			
**Prostatitis**	PMID: 38273299	Faecalibacterium	10	1.59 (1.08-2.34)	0.018	1,859	72,799	Positive association	European	IVW
		LachospiraceaeUCG004	14	1.64 (1.15-2.34)	0.007			Positive association		
		Sutterella	12	1.58 (1.14-2.19)	0.007			Positive association		
		Gastranaerophilales	9	1.47 (1.10-1.97)	0.008			Positive association		
		Methanobacteriaceae	9	0.69 (0.56-0.86)	0.001			Decreased risk		
		Erysipelatoclostridium	15	0.71 (0.55-0.93)	0.036			Decreased risk		
		Parasutterella	14	0.74 (0.57-0.96)	0.023			Decreased risk		
		Slackia	6	0.69 (0.49-0.96)	0.03			Decreased risk		
	PMID: 38369514	Sutterella	12	1.31 (1.03-1.68)	0.029	3,299	110,070	Increased morbidity	European	IVW
		Ruminococcaceae UCG010	6	1.37 (1.00-1.88)	0.049			Increased morbidity		
		Odoribacter	7	1.44 (1.04-2.00)	0.03			Increased morbidity		
		Gastranaerophilales	9	1.35 (1.12-1.64)	0.002			Increased morbidity		
		NB1n	12	1.19 (1.02-1.38)	0.026			Increased morbidity		
		Melainabacteria	10	1.27 (1.06-1.53)	0.01			Increased morbidity		
		Cyanobacteria	8	1.27 (1.02-1.58)	0.031			Increased morbidity		
		Erysipelatoclostridium	15	0.82 (0.68-0.99)	0.04			Lower risk		
		Eubacterium eligens group	6	0.69 (0.48-0.99)	0.047			Lower risk		
		Methanobacteriaceae	9	0.81 (0.69-0.95)	0.008			Lower risk		
		Methanobacteriales	9	0.81 (0.69-0.95)	0.008			Lower risk		
		Methanobacteria	9	0.81 (0.69-0.95)	0.008			Lower risk		
	PMID: 38573543	Methanobacteria	9	0.86 (0.74-0.99)	0.04	3,760	119297	Decreased risk	European	IVW
		Methanobacteriales	9	0.86 (0.74-0.99)	0.04			Decreased risk		
		Methanobacteriaceae	9	0.86 (0.74-0.99)	0.04			Decreased risk		
		NB1n	12	1.16 (1.01-1.34)	0.037			Decreased risk		
		Odoribactergenus Odoribacter	7	1.43 (1.05-1.94)	0.024			Decreased risk		
		Sutterellagenus Sutterella	12	1.33 (1.01-1.76)	0.041			Decreased risk		
	PMID: 38680919	Genus Sutterella	12	1.37 (1.09-1.71)	0.006	4,160	130,139	Increased risk	European	IVW
		Genus Holdemania	15	1.21 (1.02-1.43)	0.028			Increased risk		
		Phylum Verrucomicrobia	12	0.76 (0.58-0.98)	0.033			Negative association		
		Genus Parasutterella	14	0.84 (0.70-1.00)	0.045			Negative association		
	PMID: 36071874	Complement C4	286	1.04 (0.44-2.47)	0.039			Causal relationship	East Asia	IVW
	PMID: 38816661	HLA DR on Dendritic Cell	7	0.92 (0.80-0.99)	0.019	1,859	72,799	Protective effect	European	IVW
		HLA DR on plasmacytoid Dendritic Cell	8	0.91 (0.86-0.97)	0.006			Protective effect		
		HLA DR on myeloid Dendritic Cell	7	0.91 (0.84-0.98)	0.018			Protective effect		
	PMID: 37143736	Thyrotropin	59	0.82 (0.70-0.97)	0.018	1,859	72,799	Significantly influenced	European	IVW
		Overt hypothyroidism	18	0.85 (0.73-0.99)	0.046			Significantly influenced		

## 3 Results

### 3.1 Male infertility

Infertility is defined as the inability to achieve pregnancy following 12 months of regular unprotected intercourse, 50% of infertility cases are attributable to male factors (23). The MR studies included in this article investigate the causal relationships between MI and risk factors such as gut microbiota, cytokines, related diseases, modifiable risk factors, and other factors.

#### 3.1.1 Gut microbiota

Previous studies have demonstrated the association between gut microbes and MI but have not elucidated a causal relationship (24). The seven studies utilized various methods, including IVW, MR-Egger and maximum likelihood ratios, to evaluate the causal connection between the gut microbiota and MI risk.

The MR analyses indicated that certain microbes, including Anaerotruncus (25–28), Allisonella (27, 29), Barnesiella, Intestinibacter and Lactococcus (27) are positively associated with MI risk. In contrast, Bacteroidaceae (26, 28–30), Bacteroides (25–30), Romboutsia (27, 29) and Ruminococcaceae (Ruminococcaceae, genus NK4A2140group, genus UCG011) (26–28, 30, 31) are protective against the development of MI.

Moreover, Li TZ et al. identified the family Enterobacteriaceae and the order Enterobacteriales as being linked to a low risk of MI (29). An MR study by Xi YJ et al. indicated that Eubacterium venereum and Eubacterium rectale have protective effects on MI, whereas Eubacterium oxidoreducens contribute to MI risk (31). Using TSMR analysis, Ma S-C et al. reported that Bacteroideae, Bacteriaceae, Pasteurella, Clostridium rectalis are associated with MI (30).

#### 3.1.2 Cytokines

Zhang L et al. used MR methods such as IVW, MR-Egger and weighted median analyses to analyze the genetic association between cytokines and the risk of MI and concluded that the cytokines hepatocyte growth factor (HGF), IL-2ra, and RANTES potentially increase MI risk (32). Zou H et al. found that HGF reduced the risk of MI, and monocyte chemotactic protein 3 increased the risk of MI (25).

#### 3.1.3 Related diseases

Using MR analysis, Zhu XB et al. reported that in type 2 diabetes mellitus (T2DM) can cause ED and MI a European population (33, 34). Two MR studies showed no significant association between COVID-19 and MI (35, 36). Wang X et al. proposed that ulcerative colitis may increase the risk of MI (37). Chen X et al. ‘s results found that mood disorders and attention deficit hyperactivity disorder were positively correlated with MI, whereas obsessive-compulsive disorder was negatively associated with MI (38).

#### 3.1.4 Modifiable risk factors

Body mass index (BMI), body fat percentage, alcohol consumption and smoking are modifiable lifestyle factors linked to various health outcomes. Wentao et al. employed TSMR analyses to investigate the causal impacts of 22 diverse risk factors on MI and female infertility. Their findings indicated that BMI, body fat percentage, and alcohol consumption contribute to the risk of MI (39, 40). Greater smoking intensity was not strongly associated with MI according to MR analysis (41). The study of Chen X et al. found that coffee intake and cooked vegetable intakes increased the risk of MI (42). These insights underscore that the multifaceted interplay between lifestyle factors and health outcomes, moderate alcohol consumption, maintaining a healthy body weight and body fat, and practicing good lifestyle habits may help reduce MI risk and improve the quality of fertility.

#### 3.1.5 Other factors

Yuan et al. observed that for every unit increase in genetically predicted 25 hydroxyvitamin D (25OHD) levels, there was a corresponding decrease in the risk of MI (43). This finding underscores the potential importance of vitamin D (VD) in mitigating the risk of MI. Therefore, the clinical use of VD supplements that increase serum 25OHD levels may have implications for the prevention of MI in the general population. In addition, current MR studies have shown no or weak associations between MI and several risk factors, such as sleep traits (44), leukocyte telomere length (LTL) (45), and educational attainment (46).

The application of MR in MI is shown in **Table 1**.

### 3.2 Erectile dysfunction

ED, characterized by persistent difficulties in attaining or maintaining erections adequate for sexual intercourse, often stems from multifactorial etiologies and may signal underlying comorbidities requiring clinical assessment (19, 47). The MR studies included in this article investigate the causal relationships between ED and risk factors such as gut microbiota, cytokine, related diseases, drug targets and other factors.

#### 3.2.1 Gut microbiota

The gut microbiota may cause ED due to changes in endocrine sex hormone levels, the metabolic state of the organism and neurotransmitters (48). Using TSMR studies, Xu R et al. reported that the abundance of the genus Ruminococcaceae UCG-013 exhibited an inverse association with the risk of developing ED. Conversely, the genus Tyzzerella3, genus Erysipelotrichaceae UCG-003, genus LachnospiraceaeNC2004group, genus Oscillibacter, genus Senegalimassilia, and family Lachnospiraceae demonstrated positive associations with an increased risk of ED (49, 50). However, further research is needed to elucidate the pathogenic mechanism of the intestinal microbiota in ED.

#### 3.2.2 Cytokine

The IVW analysis of Kang Z et al. indicates that fibroblast growth factor 5, IL-22 receptor subunit alpha-1, and protein S100-A12 are associated with increased risk of ED, TNF-related activation-induced cytokine is associated with decreased risk (51). According to the study by Liu D et al., elevated levels of interferon-inducible protein-10 were found to significantly elevate the risk of ED, while higher levels of interleukin-1 receptor antagonist (IL-1RA) were observed to markedly reduce the risk of ED (52).

#### 3.2.3 Related diseases

##### 3.2.3.1 Cardiovascular disease

Cardiovascular diseases include coronary heart disease (CHD), ischemic stroke (IS), myocardial infarction, heart failure (HF), ischemic heart disease, and atrial fibrillation, among others. Several studies have elucidated the causal relationship between CVD and ED using MR analyses. For example, genetically predicted CHD and HF increase the risk of ED (53). MR analysis by Miaoyong et al. revealed a causal link between genetic susceptibility to IS, HF, and CHD and ED. Additionally, bidirectional analyses indicated that a genetic predisposition to ED did not increase the risk of CVD (54). An MR study by Zhao C et al. indicated that hypertension increased the risk of ED (55–57). The causal connection between CVD and ED has been inconsistent across multiple MR studies, and further research is needed to confirm these causal claims (54, 58, 59). These findings may inform ED prevention and intervention strategies for patients with CVD.

##### 3.2.3.2 Type 2 diabetes mellitus

ED and systemic health conditions such as metabolic syndrome (e.g., CVD and diabetes) may share many common risk factors (60). Bovijn J et al. used MR analysis to demonstrate that T2DM directly causes ED, independent of obesity and dyslipidemia (61, 62).

Furthermore, CVD, DM, and their comorbid conditions demonstrate frequent comorbidity with ED (63), likely mediated by shared pathological mechanisms such as endothelial dysfunction and chronic inflammatory cascades (64, 65). Therefore, these comorbidities should be carefully accounted for as potential confounders in MR analyses.

##### 3.2.3.3 Psychiatric disorders

The etiology of ED varies and can be organic, psychological or mixed (66). Consequently, ED is closely linked to neurological and mental health issues. Based on IVW analysis, Kai et al. suggested that psychiatric disorders, such as depression, significantly increase the incidence of ED, and genetically predicted depression plays a potential causal role in the development of ED (67–69).

##### 3.2.3.4 COVID-19

Multiple MR analyses have revealed a causal relationship between genetic susceptibility to COVID-19 and an increased risk of ED (70–72).

##### 3.2.3.5 Inflammatory bowel disease

MR analysis by Gao DW et al. did not reveal a causal connection between IBD and ED (73), but recent MR studies by Chen D et al. revealed that IBD can increase the risk of ED (74, 75).

#### 3.2.4 Drug targets

Some drug targeting MR analysis showed that drugs such as LDL receptor, lipoprotein lipase agonists and apolipoprotein C-III inhibitors were associated with reduced ED risk, while apolipoprotein B-100 inhibitors (76), atorvastatin (77) and aspirin (78) were associated with increased ED risk.

#### 3.2.5 Other factors

In addition to the above points, relevant MR studies have shown that numerous additional risk factors are associated with ED. For example, BMI, waist circumference, trunk fat mass, total body fat mass, poorer overall health scores, basal metabolic rate, stroke, smoking, snoring, insomnia, lipocalin and atorvastatin have been found to increase the risk of ED. A genetic predisposition to higher levels of sex hormone binding globulin reduces the risk of ED (79–84). In addition, there are many irrelevant factors, such as thyroid function (85), and periodontal disease (86) that are not associated with ED risk.

The application of MR in ED is shown in **Table 2**.

### 3.3 Prostate cancer

PCa remains the most prevalent malignancy in men (20), with mortality rates from metastatic PCa continuing to rise (87). Due to the complex mechanisms underlying the disease and the lack of a clearly defined optimal approach among diverse treatment options (88), exploring PCa-related risk factors is critical for refining clinical prevention and management strategies. The MR studies included in this article investigate the causal relationships between PCa and risk factors such as gut microbiota, circulatory substance, related diseases, modifiable risk factors, drug targets, leukocyte telomere length (LTL) and other factors.

#### 3.3.1 Gut microbiota

A reverse MR analysis by Xu F et al. indicated that a greater risk of PCa was associated with a decrease in the abundance of Prevotella (89). Zixin W et al. confirmed that Alphaproteobacteria has a protective effect on PCa. MVMR analysis revealed that the protective effect of Alphaproteobacteria on PCa might be driven by BMI, smoking, and drinking behaviors (90, 91). Using the Wald ratio method, Mingdong W et al. reported that the abundance of Allisonella was negatively correlated with bladder cancer and PCa incidence (92). The IVW estimates of Xie Q et al. suggested that the relative abundance of Akkermansia muciniphila and Bacteroides salyersiae may decrease the odds of PCa, whereas that of Eubacterium biforme may increase the odds of PCa (93).

#### 3.3.2 Circulatory substance

##### 3.3.2.1 Plasma microgranulin-beta

Plasma microseminoprotein-beta (MSP) is a protein secreted by prostate epithelial cells that may protect against the development of PCa. A nested case-control study using a two-sample inverse variance method to calculate MR estimates showed that plasma MSP concentrations were negatively related to PCa risk after adjusting for the concentration of total prostate-specific antigen. This study suggested that men with high levels of circulating MSP concentrations are at a lower risk of developing PCa and that MSP may play a causal protective role in PCa (94).

##### 3.3.2.2 Serum zinc, phosphorus and iron levels

The role of micronutrients in the development of urinary system tumors cannot be ignored. Using TSMR analysis, Marta et al. reported that an increase in serum zinc had a weak deleterious effect on PCa (95). Yi et al. conducted the TSMR study using pooled statistics from genome-wide association studies (GWAS) for four micronutrients and three major urologic cancer outcomes and demonstrated that each standard deviation (SD) increase in the serum zinc level increased the risk of PCa by 5.8% (96). The IVW analysis by Lin et al. indicated that for each SD increase in the serum phosphate concentration predicted by genetics, the risk of PCa increases by 19% (97). Using MR analysis, Jiacheng et al. reported that a genetically predicted increase in iron status was associated with a decrease in PCa risk and that iron has a protective effect on PCa risk. However, the mechanism by which micronutrients affect PCa needs further study (98, 99).

##### 3.3.2.3 Blood lipids

Studies have shown an association between lipid levels and PCa risk (100, 101). MR analyses by Anna I et al. revealed that the genetically predicted lipoprotein A concentration is correlated with the risk of PCa (102). Bull CJ et al. reported that higher low-density lipoprotein (LDL) and triglyceride levels increase aggressive PCa risk, although the evidence is weak (103). Shiqiang F evaluated the relationship between genetically proxied inhibition of LDL-cholesterol-lowering drug targets and PCa risk using MR methods. Genetically proxied proprotein convertase subtilisin/kexin type 9 (PCSK9) inhibition may involve biological mechanisms that reduce the risk of overall and early-onset PCa through the regulation of Lp (a) (104, 105). Shusheng et al. found an association between the effect of triglycerides on PCa risk by applying IVW, suggesting that the odds of PCa increase with elevated triglyceride levels (106). MR analysis by Nabila K et al. revealed that monounsaturated fat levels were positively associated with overall PCa risk (99).

##### 3.3.2.4 Amino acids

Cancer cells often exhibit abnormal growth and proliferation in which enhanced metabolism of amino acid substances is needed. Using TSMR, Yindan et al. demonstrated that serum aspartate levels may promote the development of PCa and breast cancer. An in-depth study of the underlying biochemical mechanisms would be valuable for the early assessment and diagnosis of these two cancers and for the development of clinical intervention strategies (107). MR analysis by Shaoxue Y et al. revealed that circulating alanine concentrations were positively associated with PCa risk and that genetically predicted alanine aminotransferase levels were inversely related to the risk of PCa (108).

##### 3.3.2.5 Red blood cells and hemoglobin

An MR study by Pin et al. provided evidence that elevated mean corpuscular volume, mean corpuscular hemoglobin, and mean corpuscular hemoglobin concentration are potentially associated with reduced risks of developing PCa (109).

##### 3.3.2.6 Circulating cytokines

Emma et al. performed analyses using methods such as TSMR and IVW and evaluated MR hypotheses in sensitivity and colocalization analyses, providing evidence of a positive correlation between the concentration of genetic proxies for macrophage inflammatory protein 1a (MIP1a) and overall PCa risk and a negative correlation between the concentration of genetic proxies for vascular endothelial growth factor and the risk of late-stage PCa (110). An MR study by Binghui L et al. suggested that long-term IL-6 levels may increase the risk of PCa, whereas long-term IL-1ra levels may reduce this risk (111).

##### 3.3.2.7 Circulating free testosterone

Two MR analyses showed that circulating free testosterone levels were related to elevated PCa risk, whereas circulating total testosterone levels showed no association with PCa risk (112, 113).

#### 3.3.3 Related diseases

Numerous studies have analyzed the potential association between other diseases and PCa risk using MR methods. For example, genetically predicted hyperthyroidism is related to a decreased risk of PCa occurrence (114). Patients with systemic lupus erythematosus have a lower risk of developing PCa (115, 116). Obstructive sleep apnea was significantly negatively associated with PCa susceptibility (117). There was a reverse causal relationship between PCa and pernicious anemia (118). The MR showed a significant association of PCa on erysipelas (119). Schizophrenia, depression and T2DM are not thought to be associated with PCa risk (120–123).

#### 3.3.4 Modifiable risk factors

##### 3.3.4.1 Obesity

The increasing prevalence of obesity globally poses a major threat to public health (124). However, current research suggests that the impact of obesity on PCa is complex. The precise pathophysiological mechanisms underlying the association between obesity and PCa incidence remain incompletely elucidated, with current scientific consensus yet to be definitively established (125, 126). A meta-analysis by Discacciati et al. demonstrated that obesity potentially reduces localized PCa risk and increases the risk of advanced PCa (127). Similarly, an MR analysis by Georgios et al. suggested that obesity increases the risk of advanced PCa (128). A meta-analysis of MR studies by Susanna et al. suggested that a genetically predicted higher adult BMI is related to a reduced risk of cancers such as PCa and breast cancer (129). Moreover, Nabila K et al. showed a negative correlation between BMI and overall PCa through TSMR (99, 130). There was no strong evidence that genetically determined metabolically unfavorable adiposity, favorable adiposity or BMI were correlated with overall PCa in the study by Aurora P-C et al. (131).

As for the conflicting results of the above studies, some believe that obesity may have different effects on PCa risk at different stages throughout the lifespan (132). The conclusion that a larger BMI and waist circumference are positively correlated with the risk of PCa mainly applies to the mid-to-late life, rather than early adulthood (133). Therefore, relevant MR studies should further clarify the effects of obesity at different time points on different developmental stages of PCa. In addition, current discrepancies in obesity-PCa associations across studies may stem from methodological limitations in adiposity assessment. The sole reliance on BMI as a clinical indicator of obesity may yield incomplete characterization of this relationship, as this metric fails to account for critical parameters such as metabolic health status and body composition metrics. Incorporating regional adiposity patterns (e.g., visceral vs. subcutaneous fat distribution) and functional adiposity biomarkers (e.g., leptin/adiponectin ratio) could better elucidate the heterogeneous biological pathways through which obesity may exert differential impacts on prostate carcinogenesis and disease progression (126). Moreover, the differences in the stages and classifications of PCa selected in different studies have led to varying results. Existing research has shown that obesity is associated with advanced or fatal PCa and reduces the risk of low-grade PCa (134), making the relationship with PCa incidence more complex.

In summary, the discrepancies among the research findings may stem from inappropriate assessment methods for obesity, variations in the stages and types of PCa selected across different studies, as well as the influence of obesity on PCa incidence being associated with distinct life stages.

##### 3.3.4.2 Smoking

Cigarette smoking can have deleterious effects on humans and increase the risk of a number of diseases. However, a definitive causal relationship between smoking and PCa has not yet been established. The meta-analysis of MR studies by Susanna et al. concluded that smoking preference was negatively related to the risk of PCa (135, 136). A European pooled study showed that smokers had a lower risk of PCa, and this finding may be attributable to detection bias. In addition, smokers have a greater risk of dying from PCa, possibly due to the direct impact of smoking, which may lead to poor treatment outcomes (137). Using MVMR analysis, Yongle et al. proposed a possible explanation for these implausible findings and showed that each additional increase in the lifetime smoking index increases the risk of PCa by 95%, suggesting a definite causal relationship between smoking and PCa risk (138).

#### 3.3.5 Drug targets

A MR study has shown that Sodium-glucose cotransporter 2 inhibitors inhibition is associated with an increased risk of PCa (139). Ding WJ et al. ‘s drug target MR study found that 3-hydroxy-3-methylglutaryl-assisted enzyme A reductase inhibitors (HMGCR) were associated with an elevated risk of PCa (140).Sun X et al., using a drug-targeted MR approach, found that genetically proxied metformin effects were associated with an increased risk of PCa (141).Sun L et al. ‘s MR study found that genetically proxied inhibition of PCSK9 was associated with reduced risk of PCa (105).The study by Yun Z. et al. provides strong evidence that the use of drugs that act on the renin-angiotensin system can reduce PCa risk (142). Ren F et al. proposed through MR analysis that genetically predicted KDEL containing 2, isoform CRA_a (KDELC2) is negatively associated with PCa. In addition, Kunitz-type protease inhibitor 2, Glutathione S-transferase P, and Cathepsin S may serve as potential therapeutic targets for PCa (143).

#### 3.3.6 Leukocyte telomere length

Telomeres play a significant role in the development and progression of cancer. Cells with longer telomere lengths have greater proliferative potential and a greater cumulative probability of mutation (144). In addition, it has been proposed that telomere shortening can cause end-to-end chromosome fusions and attenuate the DNA damage response, thereby increasing genomic instability and causing carcinogenesis (145). In conclusion, telomeres play a dual role in cancer development, and the direction of action may depend on the type of cancer and other influencing factors. Based on the GRS and MR data, Yixin et al. concluded that a shorter LTL is inversely associated with the risk of cancers such as PCa (146). Junfeng et al. conducted a study to evaluate the relative LTL in PCa patients and its correlation with aggressive disease characteristics at diagnosis and biochemical recurrence (BCR) following aggressive treatment (radical prostatectomy and radiotherapy). Employing the MR method, they found a notable association between shorter LTL and higher Gleason scores in PCa patients. Furthermore, in localized patients undergoing prostatectomy or radiotherapy, shorter LTL and genetically predicted shorter LTL have significant positive correlations with BCR risk, i.e., patients with shorter LTL have a worse prognosis (147). A recent MR study demonstrated that a genetically determined longer LTL was associated with greater PCa risk (148).

#### 3.3.7 Other factors

MR analysis revealed that many other factors, such as height (130, 149), circulating vitamin E levels (150), circulating vitamin C levels (151), circulating VD levels (152, 153), homocysteine levels (154), tryptophan (155), blood pressure (156), serum urea concentration (157), allergic diseases (158), Circulating Bilirubin Levels (159), processed meat, red meat (160), plasma phospholipid arachidonic acid concentrations (161), and circulating levels of C-reactive protein (162), are not associated with PCa risk or are weakly associated with PCa risk. Chen G et al. ‘s two-step MR analysis revealed that proinsulin functions as a suppressive factor in PCa, showing significant independence from insulin-like growth factor 1 (163).

The application of MR in PCa is shown in **Table 3**.

### 3.4 Prostatitis

According to the National Institutes of Health (NIH) classification system, prostatitis is categorized into four types: Type I (acute bacterial prostatitis), Type II (chronic bacterial prostatitis), Type III (chronic prostatitis/chronic pelvic pain syndrome, CP/CPPS), and Type IV (asymptomatic inflammatory prostatitis). Given that Type III (chronic non-bacterial prostatitis) accounts for approximately 90% of clinical cases (164), this study focuses on Type III prostatitis. Chronic prostatitis (chronic pelvic pain syndrome) is defined as pelvic pain accompanied by variable urinary symptoms and sexual dysfunction persisting for at least three months (165). Accumulated evidence confirms significant correlations between prostate inflammation development and multiple biomarkers, encompassing immune-inflammatory indicators, hormonal profiles, tumor-associated proteins, and nutritional parameters (166).The MR studies included in this article investigate the causal relationships between prostatitis and risk factors such as gut microbiota, complement C4, immune cells and thyroid function.

#### 3.4.1 Gut microbiota

The physiological functions of the host organism can be modulated by gut microbiota through their regulatory effects on multiple biological pathways, encompassing immune regulation, oxidative stress response, inflammatory modulation, and the maintenance of anabolic-catabolic equilibrium (167, 168). While direct evidence linking gut microbiota to prostate pathophysiology remains elusive, emerging research suggests that prostate health may be compromised through indirect pathological pathways, with chronic inflammatory processes likely serving as the principal mediating mechanism (169–171). In 2016, Shoskes et al. pioneered the application of MiSeq sequencing technology to delineate significant gut microbial dysbiosis in chronic nonbacterial prostatitis (CNP) patients (172). More recently, MR analyses have further advanced mechanistic insights into the gut microbiota-PCa causal axis through rigorous causal inference frameworks. According to these MR studies, the risk of prostatitis may be decreased by the presence of Methanebacteria, Methanobacteriales, Methanobacteraceae, Erysipelatoclostridium, the Eubacterium eligens group, phylum Verrucomicrobia and Parasutterella. Faecalibacterium, LachnospiraceaeUCG004, Sutterellagenus Sutterella, NB1n, Gastranaerophilales, Odoribactergenus, Odoribacter, Ruminococcaceae UCG010, Melainabacteria, genus Holdemania and Cyanobacteria play causal roles in promoting the development of prostatitis (173–176).

#### 3.4.2 Other factors

Few MR analyses have been conducted on prostatitis. However, certain risk factors, such as complement C4 (177), certain T cell subsets (178), and thyroid function (179) have been identified as having causal relationships with prostatitis. Complement C4, a pivotal component of the complement system, serves as a critical mediator in innate immunity by enabling rapid recognition and clearance of pathogenic microorganisms (180), while simultaneously reflecting systemic inflammatory activity (181). Importantly, a TSMR study recently validated a positive causal link between elevated complement C4 concentrations and chronic prostatitis pathogenesis (177).

While extensive research has established potential connections between immune cell activity and prostatitis (182, 183), the causal dynamics of specific immune populations in this inflammatory process remain undetermined. A recent investigation leveraging bidirectional MR systematically explored causal relationships between immunophenotypic characteristics and prostatitis pathogenesis. The analyses identified that particular T-cell subsets-notably CD3 + CD4 + T lymphocytes and CD3 + CD8 + T cells-demonstrated significant causal associations with elevated prostatitis risk (178).

Chronic prostatic inflammation may be modulated by endocrine hormone dysregulation or metabolic abnormalities (184). Although no studies have established direct associations between thyroid hormones and prostatitis risk, a large-scale observational investigation revealed prostate volume positively correlated with free thyroxine (FT4) levels (185). Given the potential overlap in pathogenic mechanisms underlying prostatic hypertrophy and prostatitis, Huang et al. employed MR to assess causal relationships between genetically predicted thyroid function alterations and benign prostatic disorders. Their findings demonstrated that elevated thyrotropin (TSH) concentrations and hypothyroidism development were inversely associated with risks of prostatic hypertrophy and inflammatory prostatic conditions (179).

The application of MR in prostatitis is shown in **Table 4**.

## 4 Discussion

### 4.1 Existing problems and solutions

While these MR studies advance our understanding of male reproductive disorders, several methodological limitations persist in the field. We examine these ongoing challenges specific to andrology research and propose solutions to enhance future studies.

#### 4.1.1 Multi-methodology validation

Although MR studies can suggest causal associations between risk factors and male-specific diseases, they do not reveal the underlying mechanisms of their effects. Thus, the estimated magnitude of the effect of exposure on outcomes obtained from MR analysis is not equivalent to the actual causal effect (186). It is also necessary to compare MR analyses with findings from large cohort studies or RCTs to evaluate the consistency and robustness of the evidence. Example illustrations are provided for reference:

1. Validating consistency between MR results and large-scale cohort studies.

For instance, in the manuscript section exploring the causal relationship between PCa and obesity, MR studies have reported inconsistent findings. We identified relevant prospective studies indicating that obesity during mid-to-late adulthood (but not early adulthood) showed inverse associations with localized PCa. These studies also revealed dual associations between BMI and fatal PCa - reduced risk in men with obesity during early adulthood versus increased risk in those with obesity during mid-to-late adulthood (187).

Although the effects of obesity on PCa remain complex, such cohort studies can provide longitudinal associations between obesity and PCa to verify consistency with corresponding MR findings. Therefore, MR results aligning with cohort discoveries in the manuscript may be considered relatively conclusive regarding causal relationships (though higher-level evidence remains necessary). For MR results inconsistent with cohort findings or showing methodological limitations, rigorous evaluation should be conducted regarding analytical process integrity, methodological completeness, and disease staging comprehensiveness.

2. Assessing robustness of MR causal inference using RCT evidence.

As our MR analysis suggests close associations between genetically proxied LDL-cholesterol-lowering drug targets and reduced risks of overall PCa/early-onset PCa, we referenced statins-related RCT outcomes to validate MR robustness (188). The MR approach inherently avoids confounding factors, while combining both methodologies compensates for individual limitations. This multi-level evidence integration substantially enhances result credibility.

In conclusion, researchers should not limit themselves to MR methodology alone. Concurrent collection of regional patient data for broader cross-sectional/observational studies is crucial to validate findings. Result verification constitutes an authorial responsibility rather than readers’ obligation.

Additionally, MR authors must avoid selective result presentation. Objective, rigorous, and comprehensive selection of instrumental variables and datasets should be ensured. Disease-related datasets should be comprehensively incorporated, with multivariable analyses employed to guarantee result robustness.

#### 4.1.2 European-dominant databases in prospective MR studies

Moreover, many prospective studies only use databases that include European populations, and there is a lack of relevant MR studies for Asian populations, which may lead to a lack of comprehensiveness and impact in the application of research results. Researchers can analyze large samples of data from different ethnic groups, taking into account population stratification, to achieve broader application of the research results. We humbly suggest some directions that might help resolve this difficulty:

1. Integration of population data.

We propose collaborating with Asian research institutions to conduct multicenter cohort studies, while advocating for government-supported transnational health data infrastructure development. This initiative should integrate educational, economic, and health datasets through standardized core variable definitions and establish a unified data collaboration platform with harmonized protocols (189).

To advance open science and data transparency, we recommend publicly sharing data preprocessing codes and statistical model parameters in research publications to enable reproducibility (190). This approach particularly encourages researchers to replicate and supplement findings with Asian population data. Furthermore, actively incorporating Asian-based studies (e.g., reports from China’s National Cancer Center (191)) would help counterbalance the current European-centric literature bias, thereby enhancing the reliability and generalizability of research conclusions.

2. Population stratification design.

Prospective studies should incorporate pre-stratification by race, region, and cultural background, with Asian populations further categorized into East, Southeast, and South Asian subgroups for distinct exposure-outcome analyses (192). Furthermore, sociocultural variables should be incorporated into analyses, particularly Asia-specific factors (e.g., family structure, healthcare accessibility) that may influence disease risk profiles.

These strategies will enhance the global representativeness of research findings, providing more generalizable evidence for precision medicine and public health policies.

#### 4.1.3 Survivorship bias

In addition, in male-specific disease research, when the disease of interest is associated with a risk of death, there may be survivorship bias. For example, when a long-term study of a disease is conducted, the participants in the final analysis are not a random sample because some of the study participants died earlier, which may have had some impact on the results. Researchers can identify and adjust for this bias in a variety of ways, such as using data collected in the early stages of the disease or applying weighting methods to adjust for survivorship. Nonetheless, completely eliminating survivor bias is challenging, so this issue should be carefully considered when interpreting the results of MR studies.

This paper discusses possible solutions to address this problem.

1. Integration of early cohort data: Guided by Elston’s intention-to-treat principle (193), researchers should prioritize the incorporation of longitudinal cohorts with early disease phenotypes to capture participants prior to mortality-driven attrition, thereby minimizing attrition bias. When including early cohorts (such as UK Biobank baseline data (194)), focus should be placed on incident PCa cases to avoid reliance on prevalent cases that may overrepresent indolent cancers.
2. Consider implementing genetic risk stratification: Categorize subgroups through Genetic Risk Score (GRS) stratification to identify PCa cases with accelerated progression, as their shorter disease latency periods reduce survival-related attrition, thereby potentially mitigating survival bias (195, 196).
3. Methodological adjustments: To address survivor bias in mortality-related exposure effects, researchers could implement strategies under semi-parametric additive hazard models as proposed by Vansteelandt et al. (197). This approach enables dynamic adjustment for survival selection through lifetime modeling of genetic exposure effects, rather than relying solely on cross-sectional data snapshots.

#### 4.1.4 Linkages between MR studies

The connections among several studies addressing similar research questions are weak, and some articles present contradictory views. For example, in studies exploring the correlation between LTL and PCa risk, one study showed that a shorter LTL was related to a reduced risk of PCa (146). However, other studies have suggested that a shorter LTL is detrimental to patient prognosis and that PCa patients with higher Gleason scores have shorter LTLs (147). The authors could review the relevant literature to discuss the plausibility of a potential causal connection between exposure and outcome, to interpret the results, or to suggest possible biological mechanisms (198). A lack of harmonization is observed among MR studies addressing similar research questions.

#### 4.1.5 Standardization of reporting

Recent methodological advancements emphasize the critical need for standardized reporting frameworks in MR studies. The STROBE-MR checklist provides a 32-item guideline to enhance methodological transparency (199, 200). These guidelines provide actionable resources for researchers to refine methodological rigor and enhance the translational value of causal inference studies.

### 4.2 Summary and outlook

There has been significant progress in applying MR to male-specific diseases, offering novel insights into etiological mechanisms and paving the way for innovative preventive and therapeutic strategies. The clinical translation of MR findings can directly inform actionable approaches, including: (1) lifestyle interventions (BMI, smoking, and dietary optimization) for personalized prevention; (2) microbiome-targeted therapies (probiotics/nutritional modulation) in high-risk groups; (3) drug repurposing and development guided by genetic evidence; (4) early screening protocols based on genetic risk networks (For example, CVD and T2DM can serve as early screening recommendations for high-risk populations of ED); and (5) precision therapies (e.g., anti-inflammatory agents) leveraging causal biomarker profiles.

With the increasing abundance of genomics data, the number of IVs that can be used in MR studies is increasing, and the accuracy and resolution of the studies will continue to improve. In addition, with the increase in computational power and the continuous improvement of statistical methods, more complex genetic modeling problems are expected to be solved, and the potential of MR in the study of male-specific diseases is promising.

Current MR studies on male-specific diseases should further improve the quality of study design, pay attention to the standardization of reporting and linkage with previous large MR studies, rigorously validate the results, and make appropriate adjustments for possible bias. In addition, interdisciplinary cooperation should combine expertise in genetics, epidemiology and clinical medicine and make reasonable assumptions and comprehensive interpretations of potential causality by combining the biological mechanisms of diseases and evidence from observational cohort studies in an effort to provide a valuable basis for research on the etiology of male-specific diseases as well as for the formulation of preventive policies in public health.
